# Deciphering the causal association and underlying transcriptional mechanisms between telomere length and abdominal aortic aneurysm

**DOI:** 10.3389/fimmu.2024.1438838

**Published:** 2024-08-21

**Authors:** Jiyu Zhang, Xinyi Xia, Shujie He

**Affiliations:** ^1^ Department of Cardiology, Union Hospital, Tongji Medical College, Huazhong University of Science and Technology, Wuhan, China; ^2^ Hubei Key Laboratory of Biological Targeted Therapy, Union Hospital, Tongji Medical College, Huazhong University of Science and Technology, Wuhan, China; ^3^ Hubei Engineering Research Center for Immunological Diagnosis and Therapy of Cardiovascular Diseases, Union Hospital, Tongji Medical College, Huazhong University of Science and Technology, Wuhan, China

**Keywords:** abdominal aortic aneurysm, telomere length, Mendelian randomization, bioinformatic analysis, diagnostic biomarkers

## Abstract

**Background:**

The purpose of this study is to investigate the causal effect and potential mechanisms between telomere length and abdominal aortic aneurysm (AAA).

**Methods:**

Summary statistics of telomere length and AAA were derived from IEU open genome-wide association studies and FinnGen R9, respectively. Bi-directional Mendelian randomization (MR) analysis was conducted to reveal the causal relationship between AAA and telomere length. Three transcriptome datasets were retrieved from the Gene Expression Omnibus database and telomere related genes was down-loaded from TelNet. The overlapping genes of AAA related differentially expressed genes (DEGs), module genes, and telomere related genes were used for further investigation. Telomere related diagnostic biomarkers of AAA were selected with machine learning algorisms and validated in datasets and murine AAA model. The correlation between biomarkers and immune infiltration landscape was established.

**Results:**

Telomere length was found to have a suggestive negative associations with AAA [IVW, OR 95%CI = 0.558 (0.317-0.701), P < 0.0001], while AAA showed no suggestive effect on telomere length [IVW, OR 95%CI = 0.997 (0.990-1.004), P = 0.4061]. A total of 40 genes was considered as telomere related DEGs of AAA. PLCH2, PRKCQ, and SMG1 were selected as biomarkers after multiple algorithms and validation. Immune infiltration analysis and single cell mRNA analysis revealed that PLCH2 and PRKCQ were mainly expressed on T cells, while SMG1 predominantly expressed on T cells, B cells, and monocytes. Murine AAA model experiments further validated the elevated expression of biomarkers.

**Conclusion:**

We found a suggestive effect of telomere length on AAA and revealed the potential biomarkers and immune mechanism of telomere length on AAA. This may shed new light for diagnosis and therapeutics on AAA

## 1 Introduction

Abdominal aortic aneurysm (AAA) is a chronic condition characterized by local dilation of the abdominal aorta, resulting from adverse remodeling of the arterial wall leading to permanent local enlargement (1). Once ruptured, AAA often progresses to life-threatening consequences, with a mortality rate exceeding 80% (1, 2). Multiple risk factors are involved in the occurrence and progression of AAA, including aging, smoking, hypertension, hyperlipidemia, male gender, Caucasian ethnicity, and positive family history (2, 3). Various mechanisms, such as inflammatory cells, cytokine production, matrix metalloproteinases (MMPs), smooth muscle cell phenotype switching, smooth muscle cell death, neovascularization, and thrombosis, participate in the development and progression of AAA (4–6). Currently, there are no effective drugs to prevent the growth or rupture of abdominal aortic aneurysms, and surgical or endovascular repair is the only treatment option for this disease (2). Therefore, it is crucial to explore the new pathogenesis of AAA and identify corresponding treatment targets.

Telomeres are small DNA-protein complexes located at the ends of eukaryotic linear chromosomes (7). The telomeric short repeat sequence TTAGGG, together with telomere-binding proteins, forms a special “cap” structure, which functions to maintain chromosome integrity and control the cell division cycle (8). The telomere DNA sequence is shortened by a certain amount after each cell division (7). After the telomere DNA sequence is “truncated”, the normal DNA sequence on the inner side of the telomere will be damaged, resulting in abnormal cell activity and promoting cell aging (8). Therefore, the length of telomeres reflects the cell replication history and replicative potential, known as the “mitotic clock” of cells (9). Studies have confirmed that telomere length is significantly correlated with various aging, metabolic, and tumor-related diseases (8, 10). Besides, decreased telomere length has been associated with an increased risk of cardiovascular diseases, such as hypertension and atherosclerosis (10).

As an arterial degenerative disease, aging is one of the main risk factor for AAA (11). Since telomere length emerges as a gauge of cellular ageing, therefore it is essential to explore the causal relationship and potential mechanisms between telomere length and AAA (12). Mendelian randomization (MR) studies use genetic variation as a tool to effectively control for other confounding factors, making research results more objective and improving the accuracy of causal inference. In this study, by obtaining Genome-Wide Association Studies (GWAS) data, we first confirmed the potential causal relationship between decreased telomere length and AAA prevalence using a bidirectional MR approach. Subsequently, through transcriptomic data from Gene Expression Omnibus (GEO), we obtained genes associated with both telomere length and AAA for further biomarker screening and research. Our study confirmed the causal relationship between telomere length and AAA susceptibility and elucidated its relevant molecular and immune-inflammatory mechanisms.

## 2 Methods

### 2.1 Bi-directional MR analysis

#### 2.1.1 MR study design

**Figure 1:**
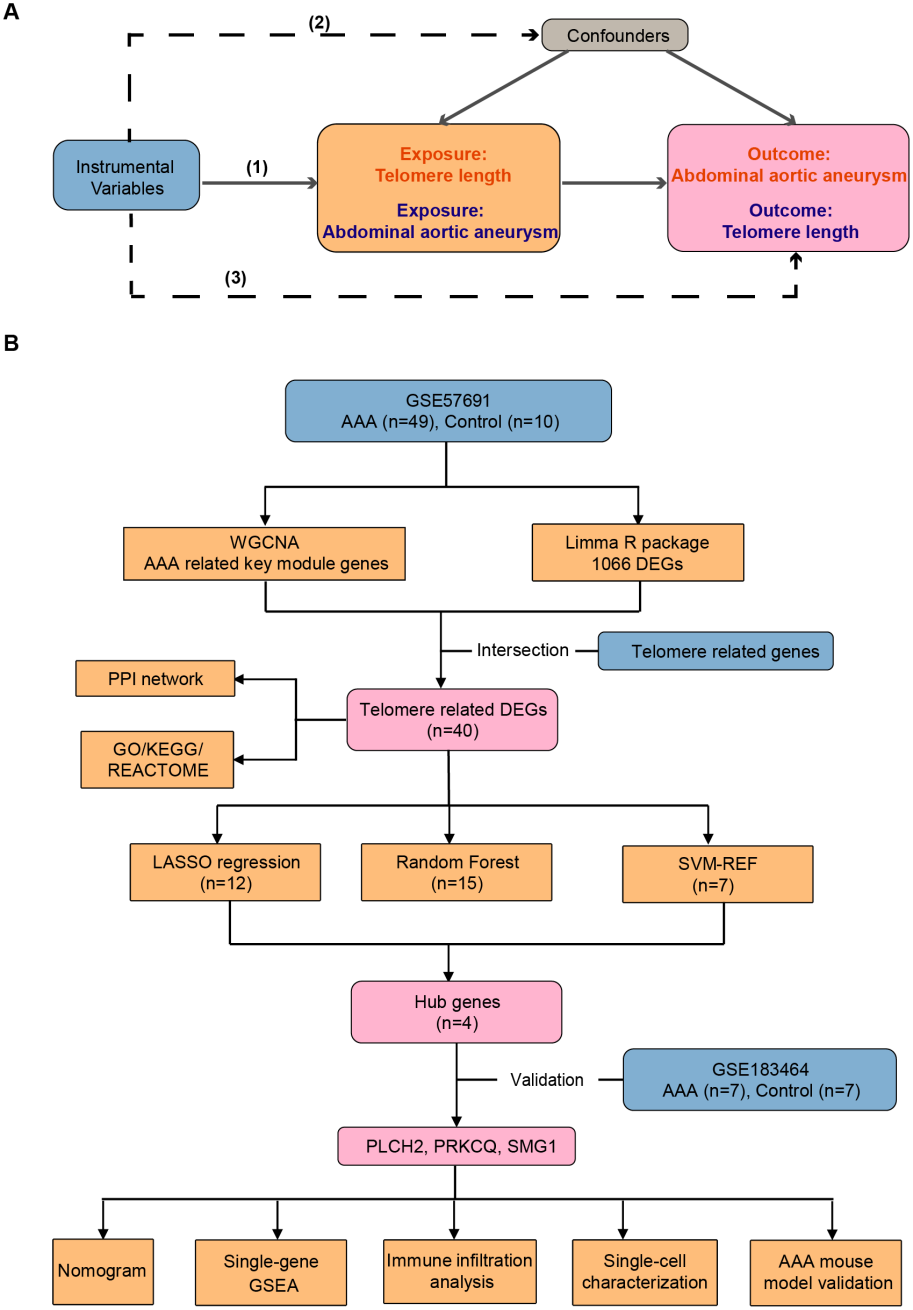
Workflow of the study. **(A)** The design of the mendelian randomization (MR) study. The MR study is grounded on three fundamental assumptions: (1) Statistical significance of instrumental variables (IVs) associated with the exposure is essential. (2) IVs should not be correlated with any known confounding factors that may distort the association between exposure and outcome. (3) IVs should only affect outcomes indirectly through the exposure pathway. Solid lines indicate relevance, while dashed lines signify irrelevance. **(B)** Identification and validation of telomere-related genes and immune profiles in abdominal aortic aneurysm. AAA, abdominal aortic aneurysm; WGCNA, weighted genes co-expression network analysis; DEGs, differentially expressed genes; PPI, protein-protein interaction; GO, gene ontology; KEGG, Kyoto Encyclopedia of Genes and Genomes; LASSO, least absolute shrinkage and selection operator; SVM-RFE, support vector machine recursive feature elimination.

The design of the MR study is depicted in **Figure 1A**. Bi-directional MR analysis was performed to explore the causal association between AAA and telomere length. MR investigations adhere to three fundamental assumptions: (1) Instrumental variables (IVs) linked to the exposure must exhibit statistical significance; (2) IVs should not be correlated with any recognized confounding factors that might potentially affect the association between exposure and outcome; (3) IVs must exert their influence solely through the exposure pathway without directly impacting the outcome.

#### 2.1.2 Data source

The GWAS summary data for AAA was obtained from the largest GWAS in the FinnGen consortium, which includes genomic information from individuals of white European ancestry. The FinnGen study is a public-private partnership that combines genotypic data from the Finnish Biobank with digital health records from the Finnish Health Registry (https://finngen.gitbook.io/documentation/). The GWAS summary data for AAA consists of 3,548 AAA patients and 288,638 healthy individuals as controls (13). The diagnosis of AAA is based on the I71 classification from the International Classification of Diseases (10^th^ revision). Telomere length related GWAS data were obtained from the IEU open GWAS (https://gwas.mrcieu.ac.uk/datasets/ieu-b-4879/), involving 472,174 individuals of European ancestry (14). The genetic variants of telomere length were updated in 2021, which contains 10,894,596 single-nucleotide polymorphisms (SNPs). The Detailed information regarding the GWAS datasets utilized in the present MR study is provided in **Supplementary Table S1**.

#### 2.1.3 IVs selection

The selection of IVs followed specific criteria: (1) For genetic variants related to telomere length, SNPs reaching the genome-wide significance threshold (P < 5E-8) were chosen. For genetic variants related to AAA, SNPs reaching a locus-wide significance threshold (P < 5E-6) were selected; (2) SNPs with undesirable characteristics (r^2 > 0.001, window size < 10,000kb) were excluded; (3) SNPs associated with the outcome (P < 5E-8) were removed to adhere to the third assumption of MR analysis; (4) Variants showing associations with potential confounding factors identified using the PhenoScanner database (15), such as atherosclerosis, smoking, and hypertension, were excluded; (5) SNPs with F-statistics > 10 were chosen to ensure their potency and avoid weak instruments in the analysis. The F-statistic was calculated using the formula: F = R^2(N-K - 1)/(K (1 - R^2)), where R^2 represents the variance in exposures explained by the genetic variance, K denotes the number of SNPs, and N indicates the sample size. Ultimately, 117 IVs associated with telomere length and 36 IVs associated with AAA were identified. Details of the IVs used in this study are provided in **Supplementary Tables S2** and **S3**.

#### 2.1.4 Statistical analysis

Statistical analyses were performed using R (version 4.2.3). MR analysis was carried out utilizing R packages “Two Sample MR” (version 0.5.6) and “MRPRESSO” (version 1.0). A significance threshold of P < 0.05 was adopted as the criterion for statistical significance. The primary estimation method for MR analysis was the Inverse Variance-Weighted (IVW) approach, supplemented by MR Egger, weighted median, simple mode, and weighted mode as secondary methods. Sensitivity analyses were conducted to ensure the robustness of the findings, including tests for heterogeneity and pleiotropy. Cochran’s Q test was utilized to identify heterogeneity, while the MR Egger intercept was employed to assess potential pleiotropy. Estimates from the MR study was presented as odds ratio (OR) alongside their respective 95% confidence interval (CI) per one standard deviation increase in exposure.

### 2.2 Transcriptome analysis

#### 2.2.1 Data source

**Figure 1B** depicts the design of the transcriptome study. Transcriptional mRNA datasets utilized in this investigation (GSE57691, GSE183464, and GSE237230) were obtained from the NCBI GEO repository (https://www.ncbi.nlm.nih.gov/geo/). GSE57961 consists of 49 human AAA tissue samples and 10 control donor aortic samples, analyzed using the Illumina HumanHT-12v4 expression BeadChip platform, and was updated in 2015. GSE183464, released in 2023, includes 14 human abdominal aortic samples (7 AAA patients and 7 controls), and was sequenced using the Illumina HiSeq4000. GSE237230 contains single-cell RNA sequencing results of AAA tissue samples from 4 AAA patient. Detailed information regarding the publicly available datasets is provided in **Table 1**. Telomere related genes were downloaded from TelNet (http://www.cancertelsys.org/telnet/; **Supplementary Table S4**) (16). TelNet is a database designed for studying telomere maintenance mechanisms in cancer cells. It maintains a list of genes reported to be involved in telomere maintenance, providing details on the type of mechanisms these genes participate in and their knockdown phenotypes. Supported by the German CancerTelSys network, TelNet is curated manually by researchers from the alliance and other scientists studying telomeres.

**Table 1:** Detailed information of transcriptional datasets used in this study.

GEO accession number	Sample size	Platform	Sample type	Note
GSE57691	49 AAA and 10 HC	GPL10558	AAA tissue	Test dataset
GSE183464	7 AAA and 7 HC	GPL20301	AAA tissue	Validation dataset
GSE237230	4 AAA	GPL24676	AAA tissue	Validation dataset

GEO, Gene Expression Omnibus; AAA, abdominal aortic aneurysm; HC, healthy control.

#### 2.2.2 Analysis of differentially expressed genes

We annotated array-based gene expression matrices using the Bioconductor R package. In instances where multiple probes corresponded to the same gene symbol, we computed the average expression. DEGs between AAA and control groups were identified using the Limma R package, with thresholds of adjusted p-values < 0.05 and log2 fold change (FC) < -1 or log2 FC > 1.

#### 2.2.3 Weighted genes co-expression network analysis

WGCNA was employed to construct gene expression modules aimed at identifying patterns associated with AAA traits (17). Initially, sample clustering trees were utilized to detect and remove outlier samples. Subsequently, genes were ranked based on the median absolute deviation of gene expression, with the top 5000 genes selected. Following this, the filtered gene expression data underwent further refinement using the goodSamplesGenes function to build an unscaled co-expression network. Soft-thresholding power (β) derived from this network was then used to compute network adjacency, resulting in the creation of a topological overlap matrix. Gene modules were generated via hierarchical clustering methods and visualized using the dynamicTreeCut function. Finally, modules were merged based on a similarity threshold of feature gene expression patterns (exceeding 0.75), and the correlation between module membership estimates and gene significance was computed.

#### 2.2.4 Functional enrichment analysis of telomere related DEGs

Telomere-related DEGs underwent functional enrichment analysis based on the Gene Ontology (GO), Kyoto Encyclopedia of Genes and Genomes (KEGG), and Reactome databases. Data processing and visualization were executed using the clusterProfiler R package (18).

#### 2.2.5 Protein-protein interactions network construction

The STRING database provides an online platform for exploring PPIs of telomere related DEGs (19). PPI network was constructed with a minimum interaction score set at 0.4, and the resulting network was exported for analysis.

#### 2.2.6 Identification of diagnostic biomarkers

To identify potential telomere-related biomarkers of AAA, machine learning algorithms including least absolute shrinkage and selection operator (LASSO) regression, random forest, and support vector machine recursive feature elimination (SVM-RFE) were utilized. Overlapped genes among these algorithms were identified and listed as potential biomarkers.

#### 2.2.7 Establishment of receiver operating characteristics curve and diagnostic nomogram

ROC curves were utilized to assess the performance of each identified biomarker derived from machine learning algorithms. The area under the ROC curve (AUC) was calculated to evaluate accuracy. The findings were validated using the GSE183464 dataset. Biomarkers with AUC values exceeding 0.7 and exhibiting statistically significant differential expression between the AAA and control groups in the GSE57691 and GSE183464 datasets were selected. A nomogram plot was generated using the rms R package for further analysis.

#### 2.2.8 Immune infiltration analysis and single-gene set analysis

We utilized the CIBERSORT R package to assess the variance in immune infiltration between the AAA and control groups by characterizing the immune cell composition in each sample (20). Subsequently, Spearman correlation analysis was employed to identify associations between the immune cell composition in AAA and the identified biomarkers. Single-gene Gene Set Enrichment Analysis (GSEA) was conducted based on GO enrichment dataset using clusterProfiler R package (18).

#### 2.2.9 Single cell mRNA analysis of AAA

GSE237230 contained four AAA tissue samples from clinical patients. The single-cell transcriptome data was processed and integrated using the Seurat R package and Harmony R package, respectively. The transcriptome of total of 1407 cells was detected. Marker genes for clusters were recognized by the Seurat FindAllMarkers function (genes at least detected in 25% of cells in target population cells, log2FC > 0.25).

#### 2.2.10 Mice

Male C57BL/6J mice aged 8 to 12 weeks were obtained from Beijing Vital River Laboratory Animal Technology Co., Ltd. (Beijing, China) and housed in the animal care facility of Tongji Medical College. The mice were maintained under standard laboratory conditions with a 12-hour light-dark cycle at 25°C. All animal experiments conducted in this study complied with the guidelines of the National Institutes of Health and were approved by the Animal Care and Utilization Committee of Huazhong University of Science and Technology.

#### 2.2.11 Murine AAA models

Male mice were used for the induction of AAA as reported (21). The procedure involved the following steps: Mice were anesthetized using 4% (vol/vol) isoflurane, then safely positioned in a supine position on a heated pad to maintain warmth throughout the surgical procedure. Abdominal fur was shaved, and a midline incision approximately 1.5 cm in length was made along the abdomen, penetrating the skin and muscle layers. Carefully, contents within the peritoneal cavity were removed to expose the retroperitoneum. In brief, the infrarenal region of the abdominal aorta was isolated under a stereomicroscope. Subsequently, a small piece of gauze soaked with 10 μL of porcine pancreatic elastase (E1250, Sigma-Aldrich; MO, USA) was applied around the aorta for 45 minutes. Similarly, mice in the sham-operated group received an equivalent duration of heat-inactivated elastase treatment. Following two rinses of the peritoneal cavity, muscle and skin layers were closed with interrupted sutures.

#### 2.2.12 Immunohistochemistry

14 days after inducing AAA, mice were euthanized, and their aortas were harvested and fixed in 4% paraformaldehyde for 24 hours before being embedded in paraffin. Paraffin sections were heated, deparaffinized in xylene, and rehydrated through a series of graded ethanol baths. Immunohistochemistry staining was performed using the primary antibody against PRKCQ (ab230971; Abcam; USA). Image analysis was conducted using ImageJ (NIH; MD, USA).

#### 2.2.13 The RNA extraction and quantitative real time polymerase chain reaction

RNA extraction from murine aortas and whole blood cells was performed using the TRIzol™ RNA extraction kit (Invitrogen, 15596026CN, CA). Subsequently, reverse transcription was carried out using the PrimeScript RT Master Mix (RR036A, Takara, Japan) to synthesize cDNA. The resulting cDNA was used as a template for RT–PCR, with the SYBR Green Master Mix (RR066A, Takara; Japan) employed. The RT–PCR protocol involved (1) initial denaturation at 95°C for 3 minutes, (2) followed by denaturation at 95°C for 3 seconds, (3) annealing at 60°C for 30 minutes, (4) repeating steps (2) to (3) for an additional 40 cycles, and (5) finally, melting from 65°C to 95°C at 0.5°C increments. mRNA expression levels were normalized to β-actin to ensure accuracy, with duplicate measurements conducted for reliability. The primer sequences for qRT-PCR in mice were as follows: PLCH2: forward 5’- TTGGTCCGCTCTACTACCTG, reverse 5’- TGGGGAGTCGATGGAAATCT; PRKCQ: forward 5’- CTTGACGCCCACATTAACA, reverse 5’- TCCGCCCAGGGAGTAGAGTTC; SMG1: forward 5’- CTGCTTCCTAACATGTAAGCC, reverse 5’- TGCCATTTCTGATCTTGTTCCAT; β-actin: forward 5’- GGCTGTATTCCCCTCCATCG, reverse 5’- CCAGTTGGTAACAATGCCATGT.

#### 2.2.14 Statistical analyses

The expression of DEGs, the proportion of immune cells between the AAA and Control groups were compared using the Kruskal-Walli’s test. Spearman correlation test evaluated the correlation between biomarkers and immune cell composition. Student’s t-test was used to assess biomarker expression in murine experiments. Statistical analyses were performed using GraphPad Prism version 9.1.0 (GraphPad Software, San Diego) and R software (version 4.2.3). Statistical significance was defined as P < 0.05.

## 3 Results

### 3.1 MR analysis

#### 3.1.1 Causal relationship between telomere length and AAA

**Figure 2:**
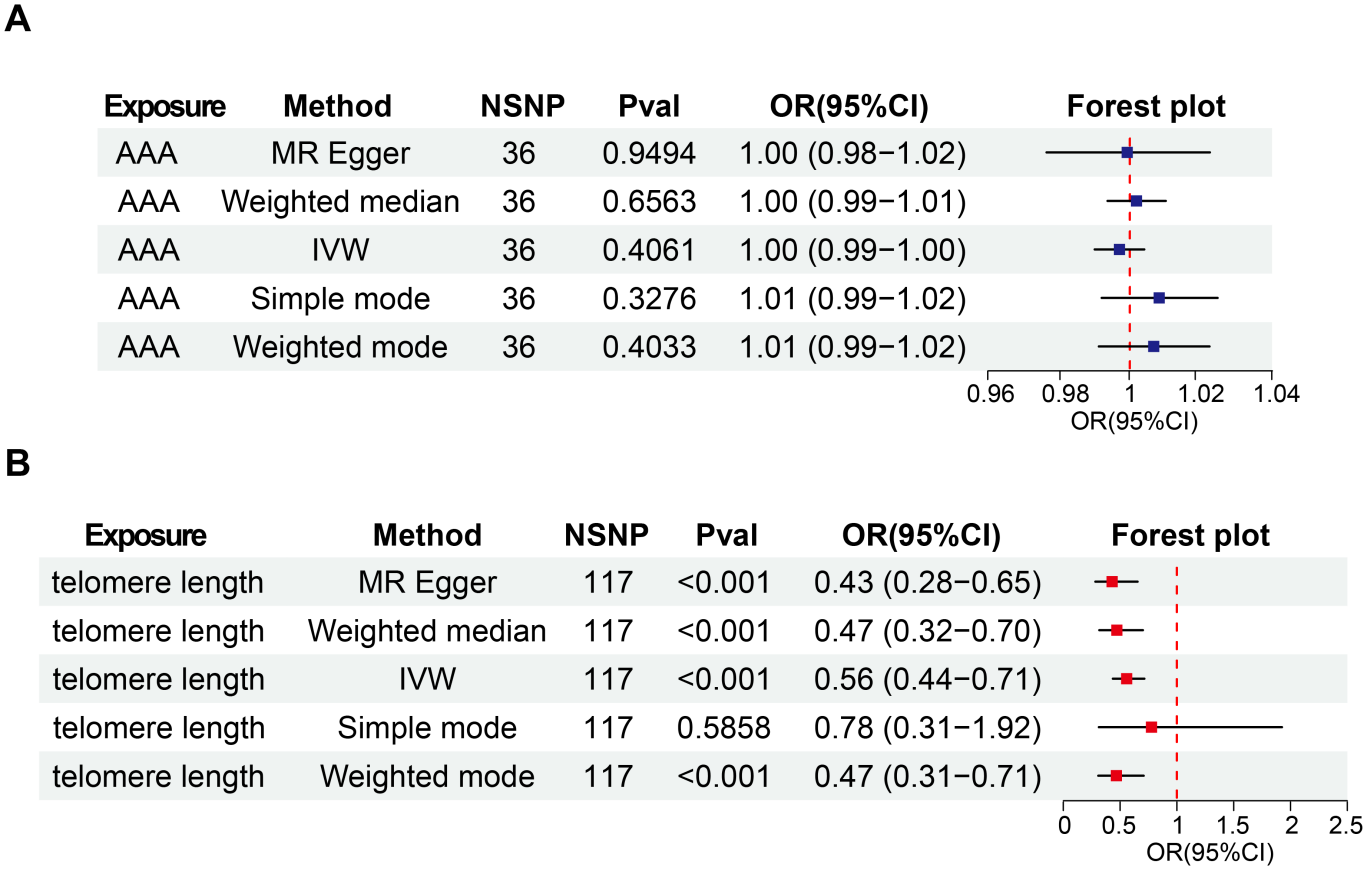
Bi-directional causal effects of AAA on telomere length **(A)** and telomere length on AAA **(B)**. OR, odds ratio; CI, confidence interval; IVW, inverse–variance weighted; AAA, abdominal aortic aneurysm; NSNP, number of SNP.

**Figure 3:**
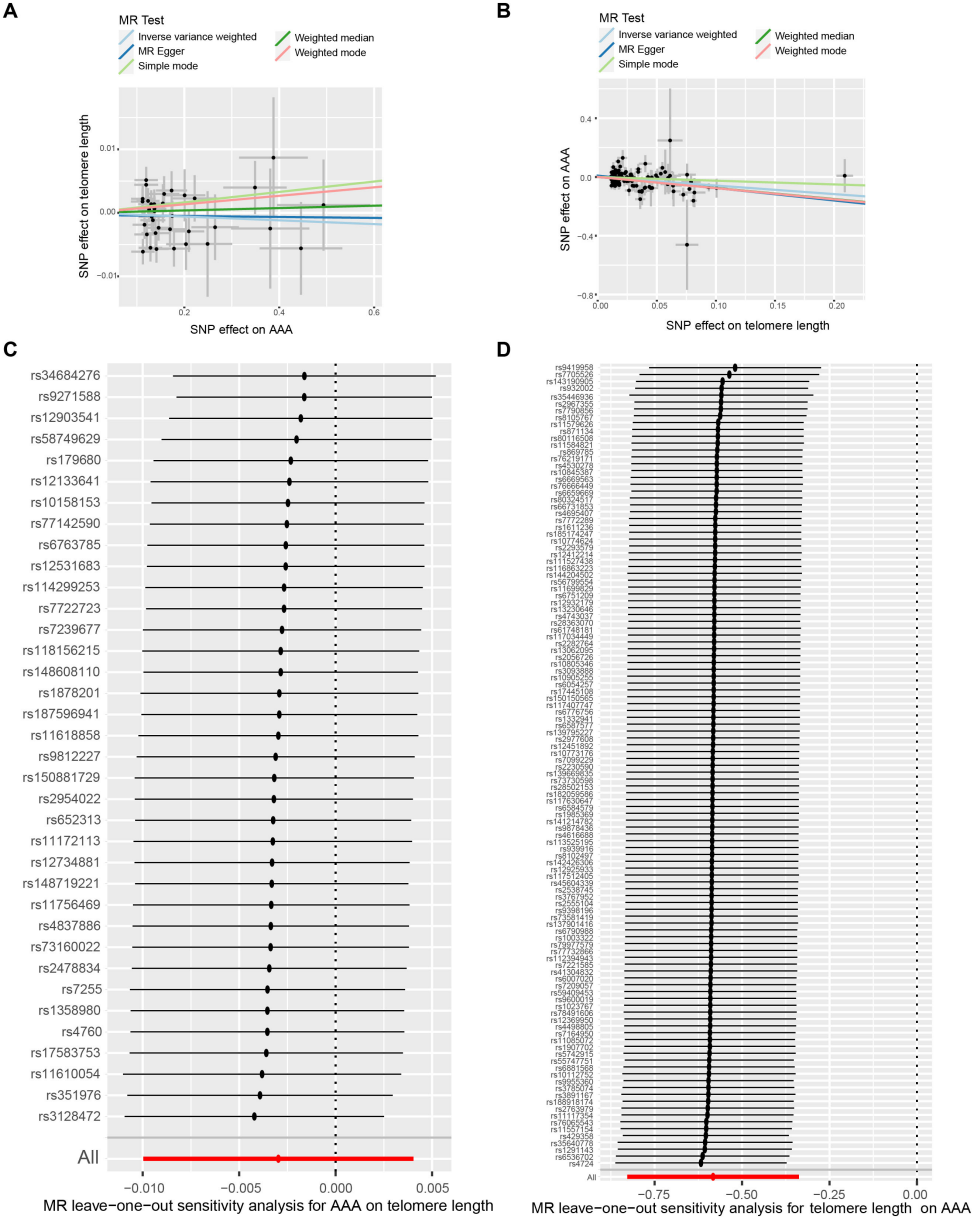
The bi-directional causality between AAA and telomere length. Scatter plots illustrate the individual estimates of the causal effects of telomere length on AAA **(A)** and AAA on telomere length **(B)**. The black dots denote SNPs, with the slope of each line representing an MR method. Leave-one-out analysis exhibits telomere length-associated SNPs correlated with AAA **(C)** and AAA-associated SNPs linked with telomere length **(D)**. AAA, abdominal aortic aneurysm; SNP, single-nucleotide polymorphisms.

In our initial analysis, we conducted a bi-directional MR investigation between AAA and telomere length. As depicted in **Figure 2A**, our findings indicate that the genetic predisposition to AAA susceptibility does not exert an effect on telomere length [IVW, OR 95%CI = 0.997 (0.990-1.004), P = 0.4061]. **Figure 2B** depicts a trend where increased telomere length is negatively correlated with AAA susceptibility, while shortened telomere length is positively correlated with AAA susceptibility [IVW, OR 95%CI = 0.558 (0.317-0.701), P < 0.001]. The scatter plots in **Figures 3A, B** demonstrate the impact of the IVs on telomere length compared to their influence on AAA, and AAA on telomere length, respectively.

#### 3.1.2 Sensitivity analysis

To ensure the reliability of our findings, we performed several sensitivity analyses. Cochran’s Q test, along with its corresponding p-value, was employed to thoroughly assess the heterogeneity of our results. Additionally, leave-one-out sensitivity analysis confirmed that no single SNP significantly influenced our findings (**Figures 3C, D**). In addressing potential pleiotropic effects, we relied on the p-value derived from the MR Egger intercept. Detailed sensitivity analysis is provided in **Supplementary Table S5**, where no evidence of pleiotropic effects was observed.

### 3.2 Transcriptome analysis

#### 3.2.1 AAA associated gene modules

**Figure 4:**
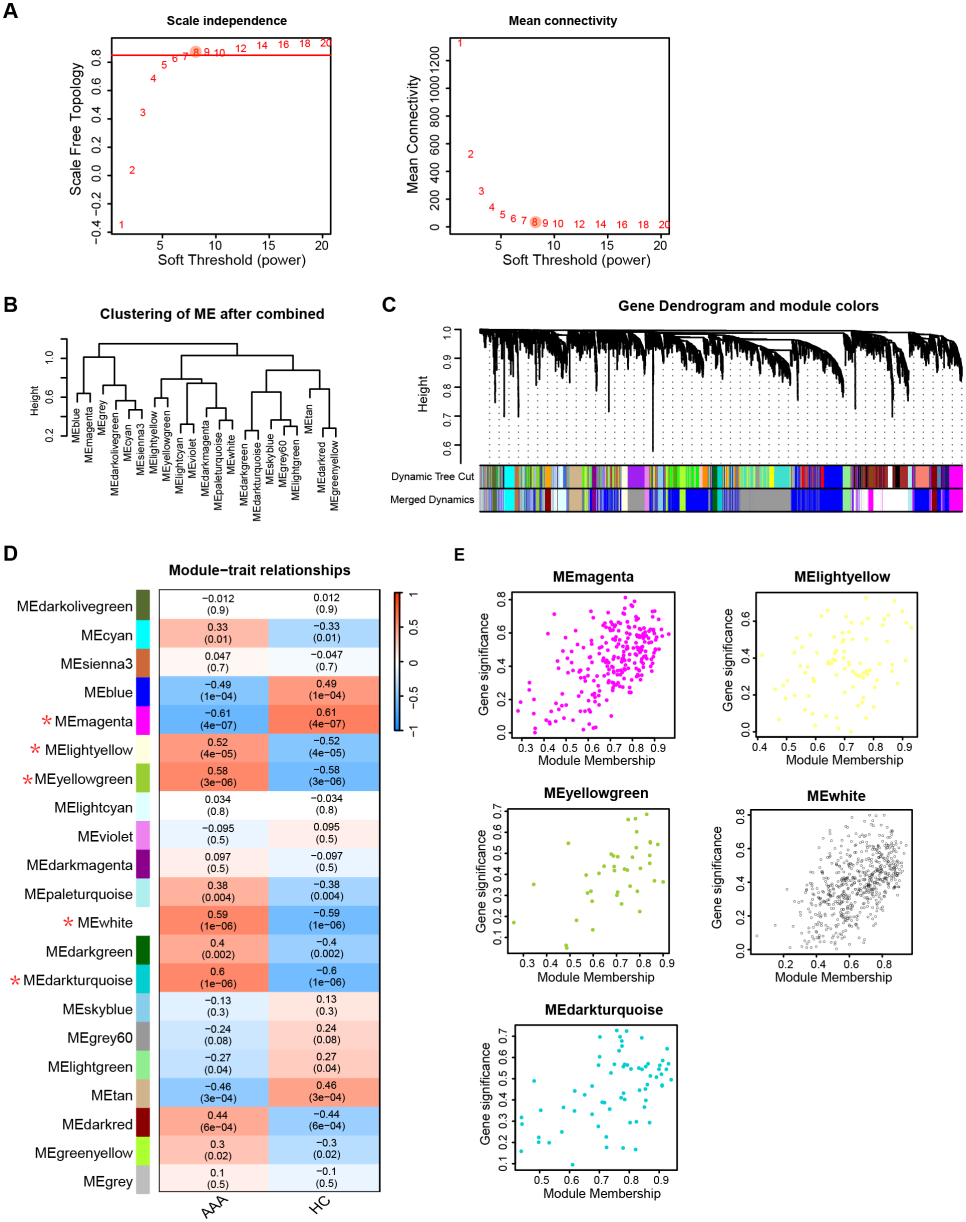
AAA associated key module genes selection. **(A)** Selection of soft threshold power (β) based on scale independence and mean connectivity. **(B)** Hierarchical clustering of gene modules after merging. **(C)** Visualization of gene dendrogram and module colors obtained through hierarchical clustering. **(D)** Heatmap illustrating the association between gene modules and AAA traits. Gene modules with a threshold of P value < 0.05 and correlation coefficients > 0.5 with AAA are marked an asterisk (*). **(E)** Correlation between module membership and gene significance of gene modules in **(D)**. AAA, abdominal arotic aneurysm; HC, healthy control.

To delve into the underlying mechanism of telomere length in AAA, we conducted the following analysis using public transcriptional data. To uncover genes linked to AAA, we utilized WGCNA. **Figure 4A** depicts that a soft threshold (β) of 8 emerged as the optimal choice, grounded on scale-free topology and average connectivity. Subsequently, employing hierarchical clustering analysis and module merging, we identified 21 gene modules (**Figures 4B, C**). The association between these gene modules and AAA is delineated in **Figure 4D**. From these, we singled out 5 gene modules for further scrutiny, employing a threshold of P < 0.05 and a correlation coefficient > 0.5. **Figure 4E** delineates the gene significance and module membership relationship of gene modules linked with AAA.

#### 3.2.2 DEGs between AAA and control

**Figure 5:**
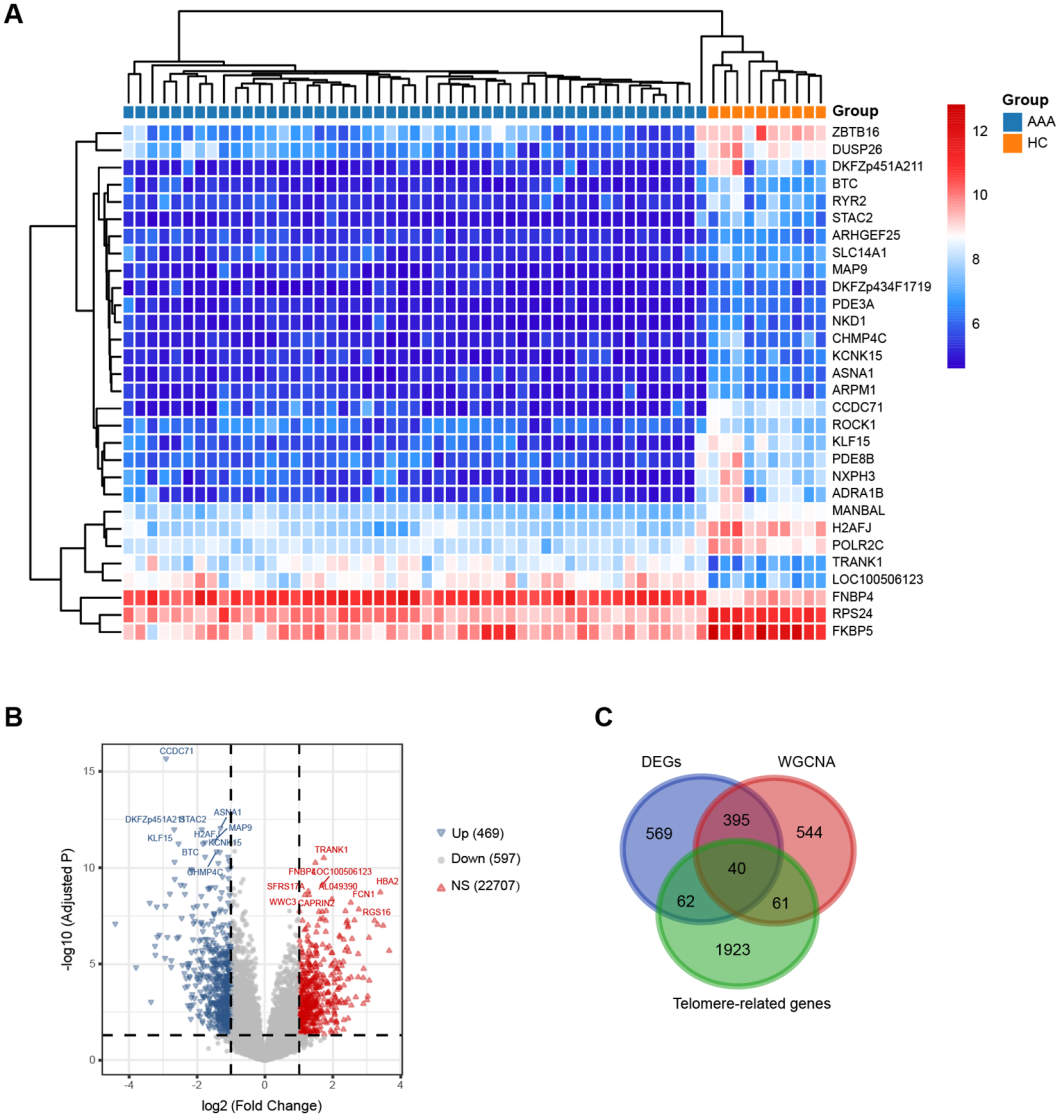
Detection of DEGs between AAA and HC. **(A)** The heatmap depicts the top 30 most significant DEGs between AAA and HC. **(B)** The volcano plot illustrates the up- and down-regulated DEGs between AAA and HC. Red triangles represent up-regulated genes (log2 FC > 1, adjusted P < 0.05), while blue triangles represent down-regulated genes (log2 FC < -1, adjusted P < 0.05). **(C)** The overlapped genes among DEGs, WGCNA-selected key module genes, and telomere-related genes. AAA, abdominal arotic aneurysm; HC, healthy control; NS, not significant; DEGs, diffrentially expressed genes.

An analysis of the DEGs between the AAA and control groups was conducted. **Figure 5A** showcases the top 30 most significant DEGs between AAA and control. A total of 1066 DEGs were identified (**Supplementary Table S6**), comprising 469 up-regulated genes and 597 down-regulated genes compared to the control group (**Figure 5B**). Additionally, we identified 40 overlapping genes at the intersection of gene modules identified by WGCNA, DEGs between AAA and control, and telomere-related genes (**Figure 5C**, **Supplementary Table S7**).

#### 3.2.3 PPI network and functional enrichment of telomere related DEGs

**Figure 6:**
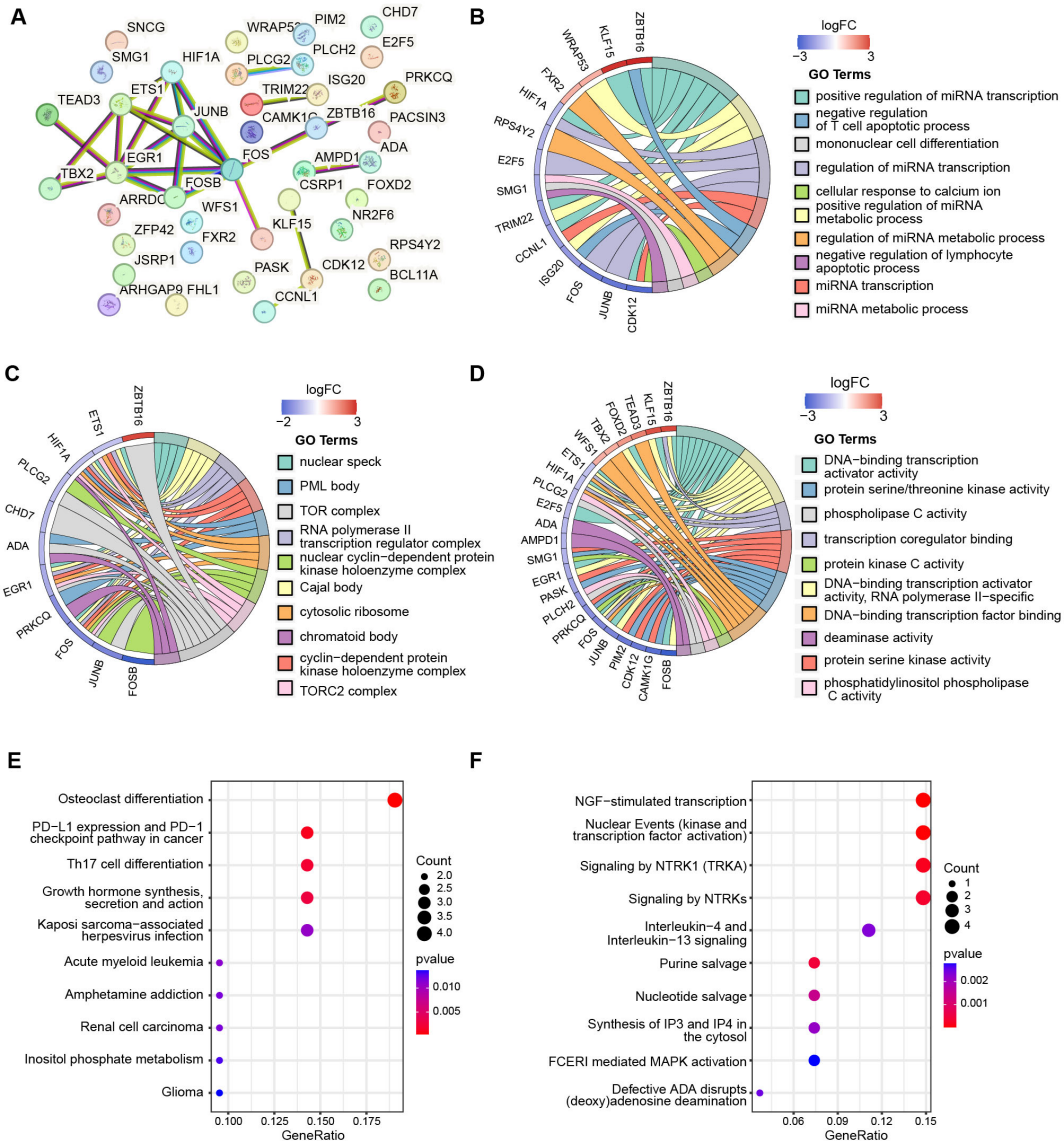
The PPI network and functional enrichment of telomere-related DEGs. **(A)** PPI network shows the interactions between 40 telomere-related DEGs. The top ten most significant enrichmemt of telomere-related DEGs based on GO categories of biological progress **(B)**, cellular components **(C)**, and molecular function **(D)**. The top ten most significant pathways of 40 telomere-related DEGs enriched with KEGG **(E)** and Reactome **(F)**.

To explore the interactions among the 40 telomere-related genes, we constructed a PPI network consisting of 40 nodes and 24 edges (**Figure 6A**). The top ten most significantly enriched pathways from GO categories, including Biological Process (BP), Cellular Component (CC), Molecular Function (MF), KEGG, and Reactome were displayed, with P-values set at < 0.05 (**Supplementary Tables S8**-**S10**). GO enrichment analysis revealed that the telomere related DEGs were mainly enriched in the following transcriptional activities: (1) BP, including positive regulation of miRNA transcription, positive regulation of miRNA metabolic process, and regulation of miRNA transcription (**Figure 6B**); (2) CC, including nuclear speck, Cajal body, and RNA polymerase II transcription regulator complex (**Figure 6C**); (3) MF, involving DNA-binding transcription activator activity, DNA-binding transcription activator activity, and RNA polymerase II-specific transcription coregulator binding (**Figure 6D**). Moreover, the top 10 most significant KEGG pathways were presented in **Figure 6E**, primarily associated with osteoclast differentiation, PD-L1 expression and PD-1 checkpoint pathway in cancer, and Th17 cell differentiation. **Figure 6F** illustrates the enrichment with the REACTOME database, predominantly involved in NGF-stimulated transcription, nuclear Events (kinase and transcription factor activation), and signaling by NTRK1.

#### 3.2.4 Selection of diagnostic biomarkers

**Figure 7:**
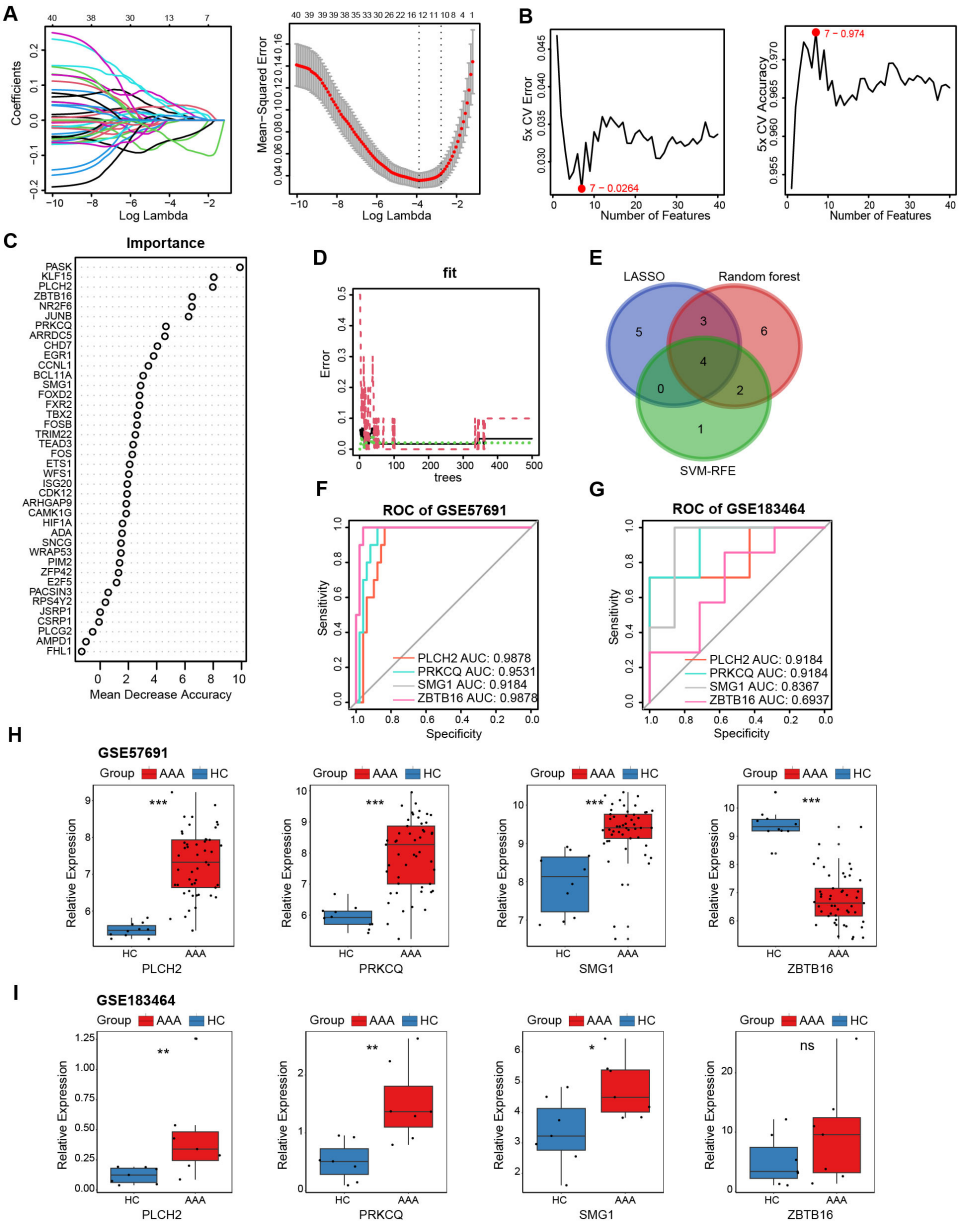
Selection and validation of AAA telomere-related biomarkers. **(A)** LASSO regression was employed to identify biomarkers with the lowest binomial deviance. **(B)** Hub genes with the highest accuracy and lowest error after 5 folds via the SVM-RFE algorithm. **(C)** Telomere-related DEGs were ranked according to importance using random forest analysis. **(D)**. The diagnostic error relating to HC, AAA, and total groups was visualized from the random forest. **(E)** Venn plot illustrates the intersection of 4 candidate biomarkers identified by the three algorithms. ROC curves generated from the 4 biomarkers in GSE57691 **(F)** and GSE183464 **(G)**. The comparison of normalized expression level of 4 biomarkers in GSE57691 **(H)** and GSE183464 **(I)**. Kruskal-Walli’s test in **(H, I)**. *P<0.05; **P<0.01; ***P<0.001; ns, not significant.

To identify key genes among the 40 telomere-related DEGs, we applied three machine learning algorithms. LASSO logistic regression identified 12 genes with the lowest binomial deviance (**Figure 7A**). Additionally, the SVM-REF approach yielded 7 genes with the lowest error and highest accuracy after 5-fold calculation (**Figure 7B**). Random Forest ranked the 40 genes based on mean decrease accuracy (**Figures 7C, D**). Following intersection, PLCH2, PRKCQ, SMG1, and ZBTB16 emerged as candidate biomarkers (**Figure 7E**). The AUC of these candidates was calculated in the GSE57691 dataset (**Figure 7F**) and the validation dataset GSE183464 (**Figure 7G**). Moreover, the expression levels of these candidates were compared in the GSE57691 dataset (**Figure 7H**) and the validation dataset GSE183464 (**Figure 7I**). After rigorous selection, PLCH2, PRKCQ, and SMG1 were selected based on their satisfactory performance criteria, exhibiting an AUC > 0.8 and statistically significant differences in expression between the AAA and control groups.

#### 3.2.5 Diagnostic nomogram construction and single-gene GSEA

**Figure 8:**
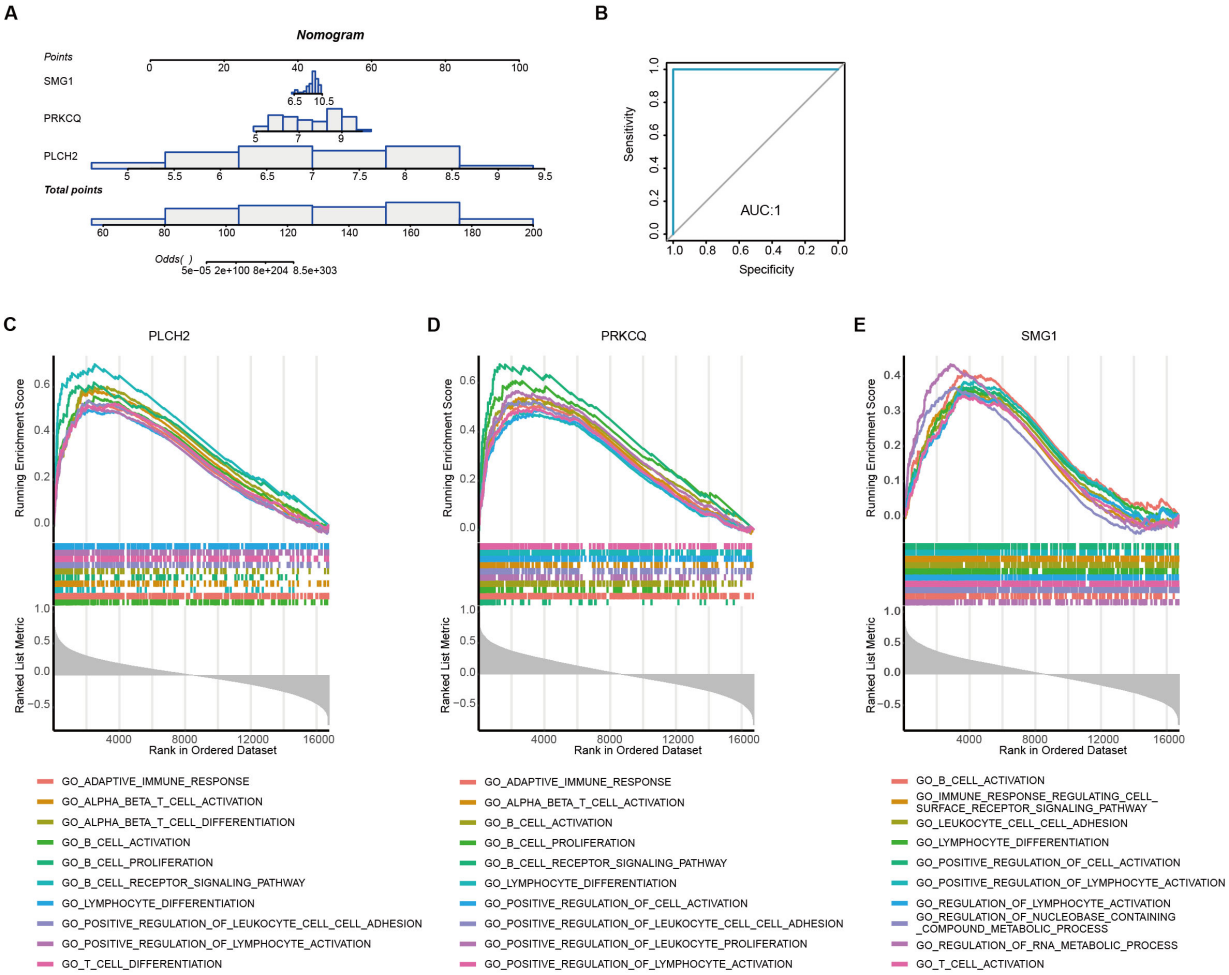
Diagnostic nomogram construction and single-gene set enrichment analysis **(A)** Establishment of the nomogram based on the expression of PLCH2, PRKCQ, and SMG1. **(B)** ROC curve derived from the nomogram. Top 10 most significant GSEA enrichment of PLCH2 **(C)**, PRKCQ **(D)**, and SMG1 **(E)** based on GO.

To assess the diagnostic robustness of telomere-related biomarkers in AAA, we constructed a nomogram based on the expression levels of PLCH2, PRKCQ, and SMG1 (**Figure 8A**). The ROC curve of the nomogram demonstrated its strong diagnostic value, with an AUC of 1 (**Figure 8B**). To further reveal the potential pathways of the telomere-related diagnostic PLCH2 (**Figure 8C**), PRKCQ (**Figure 8D**), and SMG1 (**Figure 8E**) in AAA, single-gene GSEA was conducted. Common enrichment pathways of the 3 biomarkers including “B CELL ACTIVATION”, “LYMPHOCYTE DIFFRENTIATION PATHWAY”, and “POSITIVE REGULATION OF LYMPHOCYTE ACTIVATION”, indicating the adaptative immunity activity bridges the effect of the biomarkers in AAA pathology. The top 10 most significant detailed results of single-gene GSEA based on GO were shown in **Supplementary Tables S11**-**S13**.

#### 3.2.6 Immune infiltration analysis

**Figure 9:**
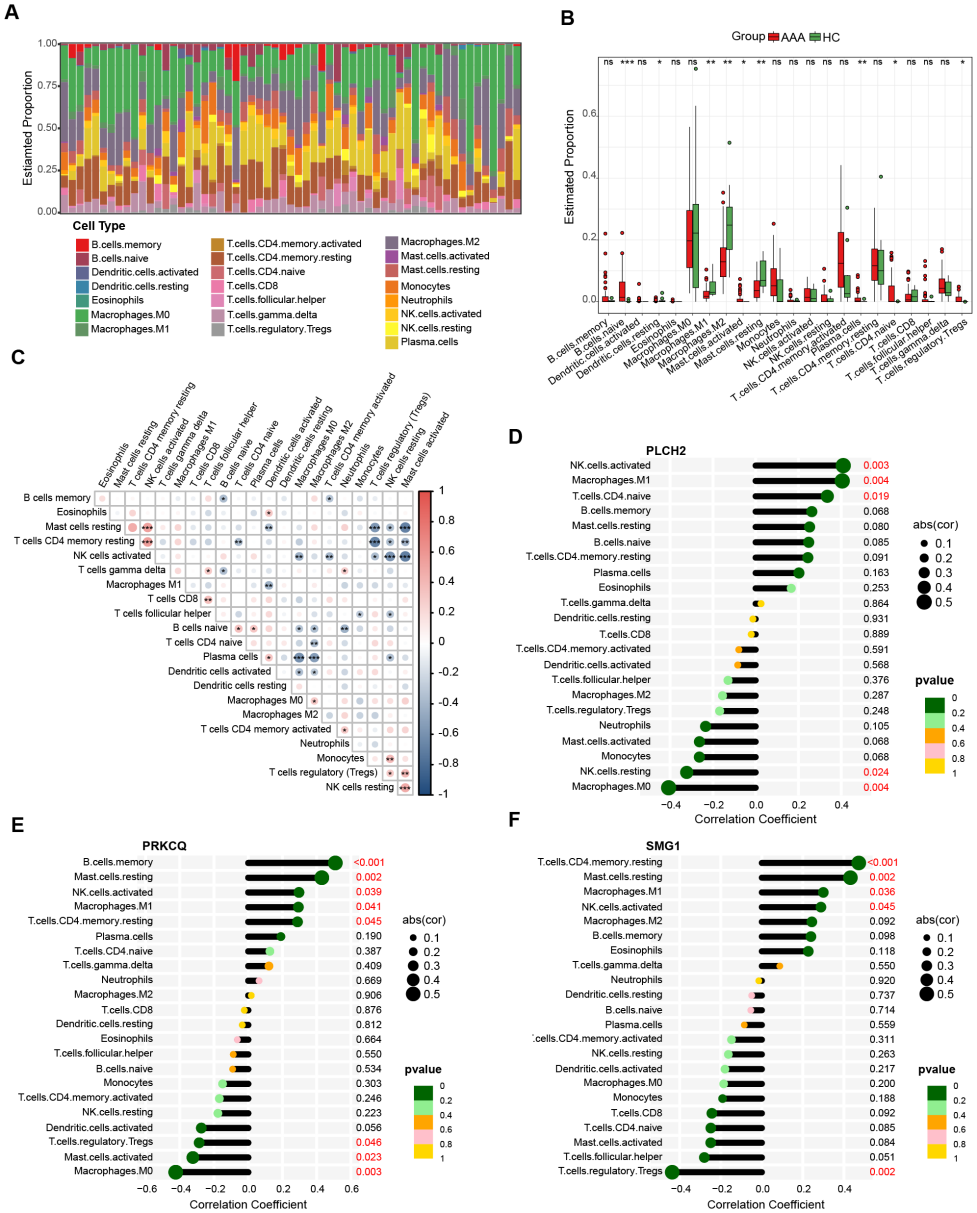
Immune infiltration analysis of AAA. **(A)** Bar plot displays the relative proportion of immune cells in AAA and control. **(B)** Differential proportion of immune cell populations in AAA and control. The association between immune cell composition in AAA samples **(C)** and their correlation with the expression levels of PLCH2 **(D)**, PRKCQ **(E)**, SMG1 **(F)**. Kruskal-Walli’s test in **(B)**, Spearman correlation analysis in **(C–F)**. *P<0.05; **P<0.01; ***P<0.001; ns, not significant.

Previous research has highlighted the crucial involvement of immune cells and inflammatory responses in AAA pathogenesis. **Figure 9A** presents the quantification of the relative proportions of 22 immune cell types in AAA, of which B cells naïve, activated Mast cells, activated memory T cells, naïve CD4+ T cells, and Tregs show elevated proportions in the AAA group, whereas M1 Macrophages, M2 Macrophages, and resting Mast cells exhibit decreased proportions compared to the control group (**Figure 9B**). Subsequently, Spearman correlation analysis was conducted to explore the relationship between immune cell composition (**Figure 9C**) and their association with the three biomarkers, namely PLCH2, PRKCQ, and SMG1 (**Figures 9D–F**). The results indicate these biomarkers exert an important role on impacting the immune cells in AAA.

#### 3.2.7 Single-cell mRNA characterization

**Figure 10:**
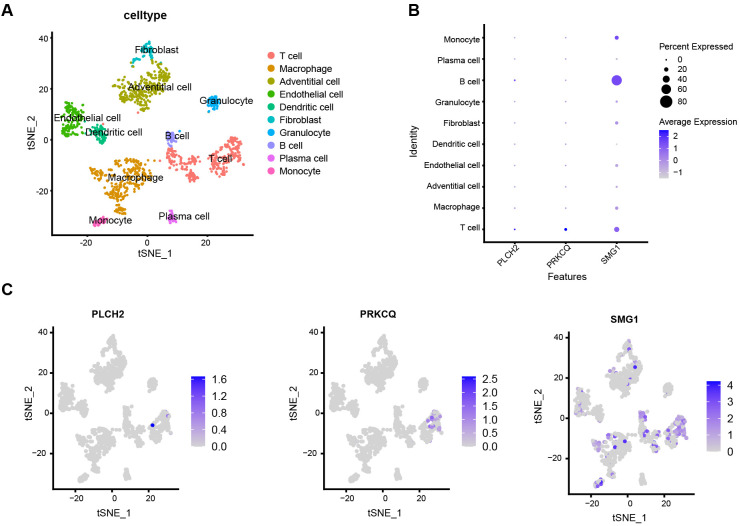
Single cell characterization. **(A)** tSNE plot shows 1407 cells from four samples diagnosed with AAA, colored by 10 major cell type clusters. **(B)** Dot plot demonstrates the relative expression of PLCH2, PRKCQ, and SMG1 in different cell clusters. **(C)** Feather plot indicates the relative expression of PLCH2, PRKCQ, and SMG1 in cell clusters of tSNE dimensionality reduction map.

To directly explore the relationship between local lesion cells of AAA and the expression of PLCH2, PRKCQ, and SMG1, we conducted single-cell mRNA analysis. The single-cell database consisted of 1407 cells from AAA local tissue samples of 4 patients. Using the tSNE algorithm, we identified and visualized 10 clusters of immune cells in the integrated dataset (**Figure 10A**). The marker genes used for annotating cell types of the ten immune cell clusters are provided in **Supplementary Table S14**. Biomarkers expression was further depicted in **Figures 10B, C**. PLCH2 and PRKCQ were predominantly expressed on T cells, whereas SMG1 was expressed across multiple clusters, including T cells, B cells, macrophages, and monocytes.

#### 3.2.8 Murine AAA model validation

**Figure 11:**
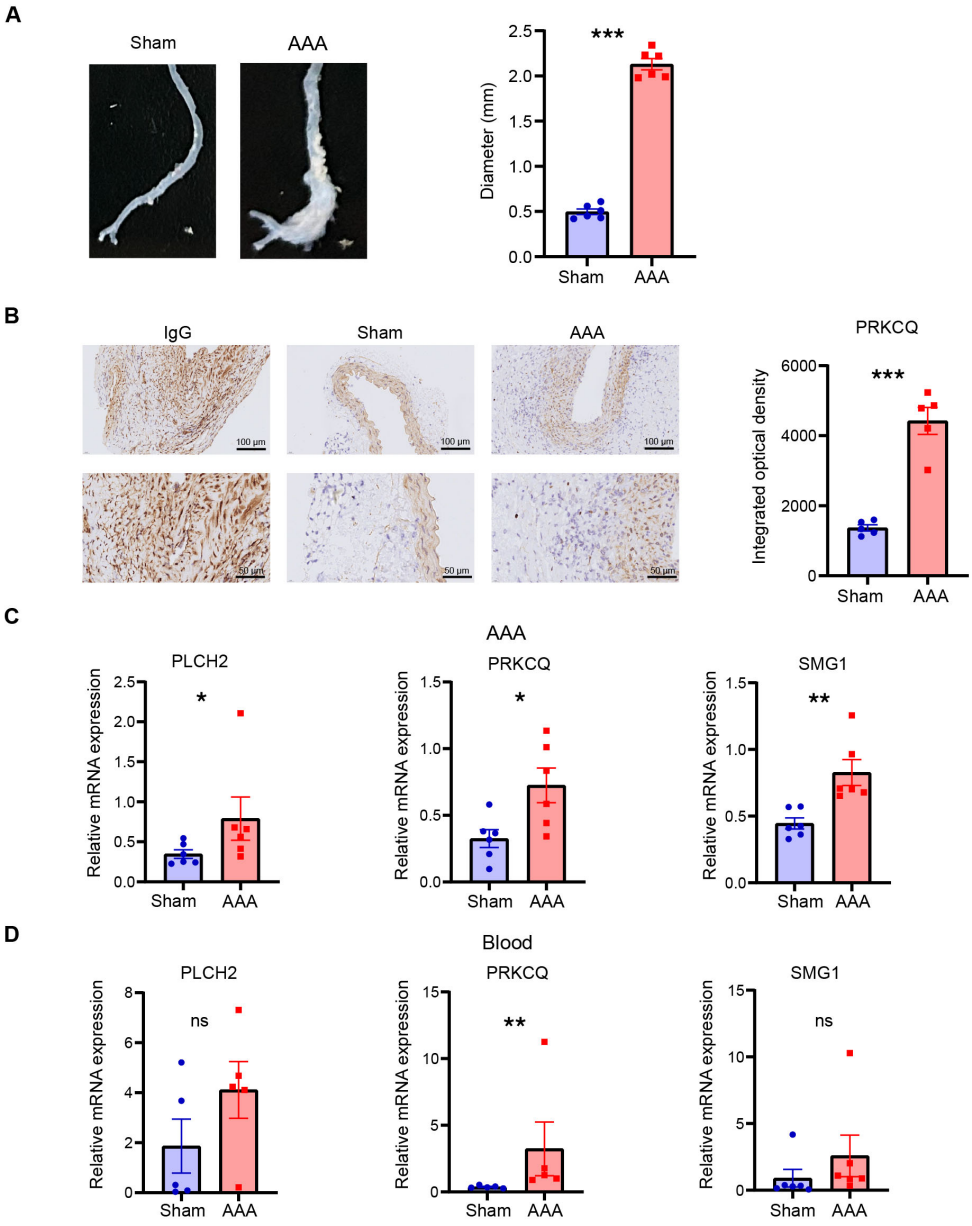
The validation of biomarkers in murine AAA model. **(A)** Representative general photos of murine in AAA group and sham group. The diameters of aortas were measured. **(B)** Immunohistochemistry of PRKCQ in aortas of AAA and sham group. Relative expression of PLCH2, PRKCQ, and SMG1 in aorta **(C)** and blood **(D)**. Students’ t test. *P<0.05; **P<0.01; ***P<0.001; ns, not significant.

To validate the expression of the telomere-related biomarkers, murine AAA model was induced to validate the expression of PLCH2, PRKCQ and SMG1 in AAA (**Figure 11A**). Immunohistochemistry of PRKCQ reveals a significant elevation in murine AAA compared with sham group (**Figure 11B**). The relative expression levels of PLCH2, PRKCQ and SMG1 in murine AAA were further assessed with qRT-PCR. The results revealed a significant elevation in the expression of PLCH2, PRKCQ and SMG1 in AAA group of aortas (**Figure 11C**) and blood (**Figure 11D**).

## 4 Discussion

As DNA-protein complexes that cap the ends of linear chromosomes, telomeres play a crucial role in preserving DNA integrity during cell division (7). Naturally, their length shortens with each cell division, serving as a marker of cellular aging (22). Aging profoundly affects the vascular system in several ways: firstly, it induces endothelial cell dysfunction, impairing their ability to respond dynamically to relaxation and contraction stimuli and predisposing them to a pro-thrombotic phenotype (23, 24). Secondly, aging prompts vascular smooth muscle cells to release inflammatory factors and modify the extracellular matrix, leading to increased vascular stiffness (25). Thirdly, it fosters the recruitment of immune cells and the secretion of inflammatory mediators during disease processes (26). Consequently, aging stands as a significant risk factor for various vascular conditions, including AAA, and aortic dissection (27).

In this study, we conducted bidirectional Mendelian randomization analysis, revealing a potential link between reduced telomere length and an increased risk of aneurysms [IVW, OR 95%CI = 0.558 (0.317-0.701), P < 0.0001]. Conversely, no association was found in the reverse direction [IVW, OR 95%CI = 0.997 (0.990-1.004), P = 0.4061]. Previous studies suggest that increased telomere attrition in endothelial and smooth muscle cells of AAA patients may result from oxidative stress-induced DNA and telomerase damage (28). Additionally, the deficiency of telomerase in bone marrow-derived cells is associated with a reduction in experimentally induced AAA formation in mice. This is because increased telomerase activity enhances the response time of inflammatory cells in AAA, further highlighting the importance of telomerase in the progression of AAA (29). This marks the first bidirectional MR analysis exploring the causal relationship between telomere length and AAA, utilizing genetic instruments to mitigate confounding factors and laying the groundwork for further mechanistic research.

To delve into the pathogenic mechanisms of telomere length-related genes in AAA and identify relevant pathways and diagnostic markers, we integrated telomere length-associated gene sets with transcriptomic data from the GEO database, yielding 40 differential genes. Enrichment analysis unveiled the involvement of these genes in microRNA (miR) function, oxidative stress, and leukocyte differentiation and apoptosis, suggesting their role in AAA progression. MiRs such as miR-29 (30), miR-21 (31), and miR-24 (32) exert significant influence on AAA by regulating extracellular matrix proteins, smooth muscle cell activity, and macrophage function (33). Oxidative stress, a key player in DNA damage and telomere shortening, also plays a critical role in AAA (34). Elevated oxidative stress disrupts inflammatory signaling, enhances MMP activity, induces smooth muscle cell apoptosis, and alters aortic wall collagen integrity (34). Moreover, inflammation and immune mechanisms are pivotal in AAA development, as activated immune cells foster an inflammatory milieu in the aortic wall, culminating in smooth muscle cell apoptosis, elastin degradation, aortic damage, and eventual rupture (35).

Through machine learning and rigorous validation procedures, we have identified PLCH2, PRKCQ, and SMG1 as diagnostic biomarkers for telomere-associated AAA, exhibiting an upward expression trend in AAA compared to the control group. The gene PLCH2 encodes the 5-bisphosphodiesterase eta-2 protein, facilitating the generation of second messenger molecules diacylglycerol (DAG) and inositol 1,4,5-triphosphate (IP3) (36). Studies suggest that PLCH2 activates intracellular Ca2+ mobilization in the endoplasmic reticulum, thereby amplifying G protein-coupled receptor (GPCR)-mediated signaling and participating in the transduction of various cellular signals, including those related to oxidative stress (37). The gene PRKCQ encodes protein kinase c theta type, a calcium-independent phospholipid and DAG-dependent serine/threonine protein kinase. PRKCQ is expressed in skeletal muscle cells, platelets, and T lymphocytes, where it orchestrates the activation of multiple transcription factors such as JUN, NFATC1, and NFATC2, exerting indispensable functions in T cell receptor (TCR) signaling (38). Crucial for T cell activation through immune synaptic translocation, PRKCQ regulates processes including activation, proliferation, differentiation, and survival (39). Dysregulation of PRKCQ activity has been linked to autoimmune diseases, inflammatory disorders, insulin resistance, type 2 diabetes, and the proliferation, migration, and invasion of cancer cells (40). Consistent with these findings, our study reveals elevated expression of PRKCQ in AAA, which stimulates an inflammatory T cell subset, contributing to disease progression. SMG1 encodes Serine/threonine protein kinase, playing a central role in nonsense-mediated decay of messenger ribonucleic acids containing premature termination codons by phosphorylating UPF1/REN1 (41). It forms transient SMG1-UPF1-eRF1-eRF3complexes with SMG8 and SMG9 (forming SMG1C protein kinase complexes) and UPF1, participating in messenger RNA surveillance and genotoxic stress response pathways (42).

Immune infiltration and single-cell transcriptome analysis have unveiled the diversity of immune cells in AAA and their correlation with telomere-related biomarkers. Our study indicates that PLCH2 and PRKCQ are predominantly expressed in T cells, while SMG1 is widely expressed in various local cells of AAA, including T cells, monocyte macrophages, and endothelial cells. Single-gene GSEA analysis suggests that PLCH2, PRKCQ, and SMG1 are upregulated in adaptive immunity and closely associated with T cell activation. AAA is characterized by the infiltration of inflammatory cells, extracellular matrix degradation, and dysfunction of smooth muscle cells, linked to the infiltration of inflammatory cells in the adventitia and intima of blood vessels (1, 6). These factors collectively contribute to vascular remodeling and weakening of the aortic wall. Vascular inflammation in AAA involves the chemotaxis of inflammatory cells and the release of pro-inflammatory factors, triggering a cascade of inflammatory responses. Immune cells implicated in AAA include macrophages, neutrophils, mast cells, natural killer cells, dendritic cells, B cells, and T cells, with CD4+ T helper cells playing a predominant role (43).

In this study, we initially employed a bidirectional MR approach to uncover an association between telomere shortening and increased incidence of AAA. Subsequently, leveraging public transcriptomic data, we elucidated the inflammatory immune mechanisms underlying the impact of telomere genes on AAA, identified relevant diagnostic genes and intervention targets, and further validated them in a mouse model of AAA. However, our study still has certain limitations. Firstly, in the MR study, telomere length was not refined at the cellular level. Given the local infiltration of various immune-inflammatory cells in AAA, subsequent data on telomere length SNPs targeting multiple immune cells are necessary. Secondly, in the analysis of AAA transcriptomic data, although GSE57691 includes 10 control arterial specimens and 49 tissue specimens from abdominal aortic aneurysms, peripheral blood-related transcriptomic expression data from a large sample size are also necessary to achieve diagnostic efficacy. Thirdly, in our mouse AAA model, we validated the expression of diagnostic biomarkers PLCH2, PRKCQ, and SMG1. However, further elucidation of the immune-inflammatory mechanisms and intervention targets requires additional functional experiments for confirmation.

## 5 Conclusion

This study demonstrates a causal relationship between telomere shortening and increased susceptibility to AAA. Transcriptomic data analysis identified adaptive immune responses, oxidative stress, and transcriptional regulation as playing significant roles in the impact of telomeres on AAA. Moreover, the correlation analysis of diagnostic biomarkers PLCH2, PRKCQ, and SMG1 with AAA immune inflammation further indicates the crucial role of upregulated adaptive immune responses and T cell activation. Our research elucidates the causal relationship and pathogenic mechanisms of telomere-related AAA, providing new insights for clinical diagnosis and intervention of AAA.

## Data Availability

The datasets presented in this study can be found in online repositories. The names of the repository/repositories and accession number(s) can be found in the article/**Supplementary Material**.
