# Correction: Deciphering the causal association and underlying transcriptional mechanisms between telomere length and abdominal aortic aneurysm

**DOI:** 10.3389/fimmu.2025.1734582

**Published:** 2025-11-17

**Authors:** Jiyu Zhang, Xinyi Xia, Shujie He

**Affiliations:** 1 Department of Cardiology, Union Hospital, Tongji Medical College, Huazhong University of Science and Technology, Wuhan, China; 2 Hubei Key Laboratory of Biological Targeted Therapy, Union Hospital, Tongji Medical College, Huazhong University of Science and Technology, Wuhan, China; 3 Hubei Engineering Research Center for Immunological Diagnosis and Therapy of Cardiovascular Diseases, Union Hospital, Tongji Medical College, Huazhong University of Science and Technology, Wuhan, China

**Keywords:** abdominal aortic aneurysm, telomere length, Mendelian randomization, bioinformatic analysis, diagnostic biomarkers

An incorrect number was provided for “National Natural Science Foundation of China”. The correct number is “No. 82300335”.

The original version of this article has been updated.

